# Song lyrics have become simpler and more repetitive over the last five decades

**DOI:** 10.1038/s41598-024-55742-x

**Published:** 2024-03-28

**Authors:** Emilia Parada-Cabaleiro, Maximilian Mayerl, Stefan Brandl, Marcin Skowron, Markus Schedl, Elisabeth Lex, Eva Zangerle

**Affiliations:** 1 Multimedia Mining and Search Group, Institute of Computational Perception, Johannes Kepler University Linz, Linz, Austria; 2 Department of Music Pedagogy, Nuremberg University of Music, Nuremberg, Germany; 3 AI Lab, Human-centered AI Group, Linz Institute of Technology, Linz, Austria; 4 Department of Computer Science, University of Innsbruck, Innsbruck, Austria; 5 Austrian Research Institute for Artificial Intelligence, Vienna, Austria; 6 Graz University of Technology, Graz, Austria

**Keywords:** Music, Song lyrics, Analysis, Popularity, Genre, Computational science, Computer science

## Abstract

Music is ubiquitous in our everyday lives, and lyrics play an integral role when we listen to music. The complex relationships between lyrical content, its temporal evolution over the last decades, and genre-specific variations, however, are yet to be fully understood. In this work, we investigate the dynamics of English lyrics of Western, popular music over five decades and five genres, using a wide set of lyrics descriptors, including lyrical complexity, structure, emotion, and popularity. We find that pop music lyrics have become simpler and easier to comprehend over time: not only does the lexical complexity of lyrics decrease (for instance, captured by vocabulary richness or readability of lyrics), but we also observe that the structural complexity (for instance, the repetitiveness of lyrics) has decreased. In addition, we confirm previous analyses showing that the emotion described by lyrics has become more negative and that lyrics have become more personal over the last five decades. Finally, a comparison of lyrics view counts and listening counts shows that when it comes to the listeners’ interest in lyrics, for instance, rock fans mostly enjoy lyrics from older songs; country fans are more interested in new songs’ lyrics.

## Introduction

We are surrounded by music every day; it is pervasive in today’s society^1^ and serves many functions. For instance, people listen to music to relieve boredom, fill uncomfortable silences, foster social cohesion and communication, or regulate their emotions^2–4^. When it comes to listeners liking or disliking a track, the most salient components of music, alongside the ability of a song to evoke emotion and the singing voice, are a song’s lyrics^5^. Likewise, the interplay between melody and lyrics is imperative as lyrics have been shown to influence the emotional valence of music; particularly, lyrics can enhance the negative emotion in angry and sad music^6^. Music containing lyrics has also been shown to activate different regions in the brain compared to music without lyrics^7^.

Seen from a different angle, lyrics can be considered a form of literary work^8^. Usually written in verse form, lyrics use poetic devices such as rhyme, repetition, metaphors, and imagery^9^, and hence can be considered similar to poems^8^. This is also showcased by Bob Dylan winning the Nobel Prize in literature in 2016 “for having created new poetic expressions within the great American song tradition”^10^. Just as literature can be considered a portrayal of society, lyrics also provide a reflection of a society’s shifting norms, emotions, and values over time^11–15^.

To this end, understanding trends in the lyrical content of music has gained importance in recent years: computational descriptors of lyrics have been leveraged to uncover and describe differences between songs with respect to genre^16,17^, or to analyze temporal changes of lyrics descriptors^11,17,18^. Lyrical *differences between genres* have been identified by Schedl in terms of repetitiveness (rhythm & blues (R&B) music having the most repetitive lyrics and heavy metal having the least repetitive lyrics) and readability (rap music being hardest to comprehend, punk and blues being easiest)^16^. In a study of 1879 unique songs over three years (2014–2016) across seven major genres (Christian, country, dance, pop, rap, rock, and R&B), Berger and Packard^19^ find that songs with lyrics that are topically more differentiated from its genre are more popular in terms of their position in the Billboard digital download rankings. Kim et al.^20^ use four sets of features extracted from song lyrics and one set of audio features extracted from the audio signal for the tasks of genre recognition, music recommendation, and music auto-tagging. They find that while the audio features show the largest and most consistent effect sizes, linguistic and psychology inventory features also show consistent contributions in the investigated tasks.

Studies investigating the *temporal evolution of lyrics* predominantly focus on tracing emotional cues over the years. For instance, Dodds et al.^17^ identify a downward trend in the average valence of song lyrics from 1961 to 2007. Napier and Shamir^21^ investigated the change in sentiment of the lyrics of 6150 Billboard 100 songs from 1951 through 2016. They find that positive sentiments (e.g., joy or confidence) have decreased, while negative sentiments (e.g., anger, disgust, or sadness) have increased. Brand et al.^13^ use two datasets containing lyrics of 4913 and 159,015 pop songs, spanning from 1965 to 2015, to investigate the proliferation of negatively valenced emotional lyrical content. They find that the proliferation can partly be attributed to content bias (charts tend to favor negative lyrics), and partly to cultural transmission biases (e. g., success or prestige bias, where best-selling songs or artists are copied). Investigating the lyrics of the 10 most popular songs from the US Hot 100 year-end charts between 1980 and 2007, DeWall et al.^18^ find that words related to oneself (e.g., me or mine) and words pointing to antisocial behavior (e.g., hate or kill) increased while words related to social interactions (e.g., talk or mate) and positive emotions (e.g., love or nice) decreased over time.

Alongside changes in emotional cues, Varnum et al.^11^ find that the *simplicity* of lyrics in pop music increased over six decades (1958–2016). Similarly, Choi et al.^9^ study the evolution of lyrical complexity. They particularly investigate the concreteness of lyrics (concreteness describes whether a word refers to a concrete or abstract concept) as it has been shown to correlate with readability and find that concreteness increased over the last four decades. Furthermore, there is also a body of research investigating the evolution of lyrical content (i.e., so-called themes). For instance, Christenson et al.^22^ analyzed the evolution of themes in the U.S. Billboard top-40 singles from 1960 to 2010. They find that the fraction of lyrics describing relationships in romantic terms did not change. However, the fraction of sex-related aspects of relationships substantially increased.

Studies on the temporal evolution of music have also looked into *temporal changes of acoustic descriptors*, beyond lyrics. Interiano et al.^23^ investigate acoustic descriptors of 500,000 songs from 1985 to 2015. They discover a downward trend in “happiness” and “brightness”, as well as a slight upward trend in “sadness”. They also correlate these descriptors with success and find that successful songs exhibit distinct dynamics. In particular, they tend to be “happier”, more “party-like”, and less “relaxed” than others.

Despite previous efforts to understand the functions, purposes, evolution, and predictive qualities of lyrics, there still exists a research gap in terms of uncovering the complete picture of the complex relationships between descriptors of lyrical content, their variations between genres, and their temporal evolution over the last decades. Earlier studies focused on specific descriptors, genres, or timeframes, and most commonly investigated smaller datasets. In this paper, we investigate the (joint) evolution of the complexity of lyrics, their emotion, and the corresponding song’s popularity based on a large dataset of English, Western, popular music spanning five decades, a wide variety of lyrics descriptors, and multiple musical genres. We measure the popularity of tracks and lyrics, where we distinguish between the listening count, i. e.,  the number of listening events since the start of the platform, and the lyrics view count, i. e.,  the number of views of lyrics on the Genius platform (https://genius.com). Thereby, we investigate the following research questions in this paper: (RQ1) Which trends can we observe concerning pop music lyrics across the last 50 years, drawing on multifaceted lyrics descriptors? We expect that descriptors that correlate more strongly with the release year lead to better-performing regression models. (RQ2) Which role does the popularity of songs and lyrics play in this scenario? We expect that lyric views vary across genres, and these variations can be attributed to changes in lyrics over time.

Our exploratory study differs from existing studies in several regards: (1) we provide the first *joint analysis* of the evolution of multiple lyrics descriptors and popularity, (2) we investigate a *multitude of lyrics descriptors* capturing lyrical complexity, structure, and emotion, (3) we provide an *in-depth analysis* of these descriptors’ evolution, not only over *time* but focusing on different *genres*, and (4) we leverage a substantially *larger dataset* than most existing works.

For our analyses, we create a dataset containing 353,320 English song lyrics from the Genius platform (https://genius.com/), spanning five decades (1970–2020) in terms of the songs’ release years. Based on this collection of lyrics, we extract a wide variety of lyrics’ descriptors and popularity data for each song. In particular, we extract lexical, linguistic, structural, rhyme, emotion, and complexity descriptors and focus on five genres: rap, country, pop, R&B, and rock, as these are the most popular genres according to the widely used music streaming platform last.fm (https://www.last.fm/)^24–27^, disregarding genres for which lyrics are less frequent (e. g., jazz and classical music). Our analysis is based on two complementary analyses, as shown in Fig. 1. In our first analysis, we are interested in the evolution of pop music lyrics and the impact of descriptors in a regression task (i.e., we aim to find the predictors that are best suited to model a linear trend in a release year regression task). The second set of analyses investigates the relationship between lyrics view count, descriptors, and corresponding songs’ release year in a multiple linear regression analysis. Assessing lyrics’ view count, besides the typically analyzed measure listening play count, enables us to take into account another perspective of music popularity. In particular, lyrics’ view count allows us to expressively investigate the role played by lyrics in music consumption patterns over time (through the songs’ release year) as well as to relate such patterns with the lyrics characteristics (through the lyrics’ descriptors). Note that while listening play counts do not give any information about a listener’s interest in the lyrics, lyrics’ view count is a clear indicator of lyrics’ importance, which might not necessarily relate to a musical genre’s general popularity.

## Methods

**Figure 1:**
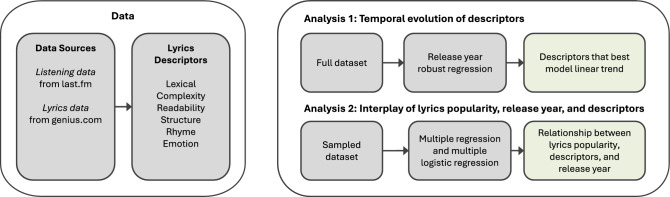
Overview of data and analyses performed. Based on a wide variety of descriptors capturing the lyrical characteristics from listening data and lyrics content, we perform two analyses. Analyses 1 identifies descriptors that are characteristic of the release year and genre. Analyses 2 investigates the relationship between the identified lyrics descriptors, popularity (listening counts and lyrics view counts), and release year.

### Data

#### Basic dataset and lyrics acquisition

For gathering song lyrics, we rely on the *LFM-2b dataset*^28^ (http://www.cp.jku.at/datasets/LFM-2b) of listening events by last.fm users, since it is one of the largest publicly available datasets of music listening data. Last.fm is an online social network and streaming platform for music, where users can opt-in to share their listening data. It provides various connectors to other services, including Spotify, Deezer, Pandora, iTunes, and YouTube, through which users can share on last.fm what they are listening to on other platforms (https://www.last.fm/about/trackmymusic). While last.fm services are globally available, their user base is unevenly distributed geographically, with a strong bias towards the United States, Russia, Germany, United Kingdom, Poland, and Brazil. In fact, an analysis of a representative subset of last.fm users found that the users from these six countries comprise more than half of last.fm’s total user base^29^.

The LFM-2b dataset contains more than two billion listening records and more than fifty million songs by more than five million artists. We enrich the dataset with information about songs’ release year, genre, lyrics, and popularity information. For quantifying the popularity of tracks and lyrics, we distinguish between the listening count, i. e.,  the number of listening events in the *LFM-2b dataset*, and lyrics view count, i. e.,  the number of views of lyrics on the Genius platform (https://genius.com). Release year, genre information, and lyrics are obtained from the Genius platform. Genres are expressed by one primary genre and arbitrarily many additional genre tags. Lyrics on the Genius platform can be added by registered members and undergo an editorial process for quality checks. We use the polyglot package (https://polyglot.readthedocs.io/) to automatically infer the language of the lyrics and consider only English lyrics. Adopting this procedure, we ultimately obtain complete information for 582,759 songs.

#### Lyrics style and emotion descriptors

Following the lines of previous research in the field of lyrics analysis^30–32^, we characterize lyrics based on stylistic (including descriptors of lexical, complexity, structure, and rhyme characteristics) and emotion descriptors. Lexical descriptors include, for instance, unique token ratio, repeated token ratio, pronoun frequency, line count, or punctuation counts, but also measures of lexical diversity^33,34^ as these have been shown to be well-suited markers for textual style^35^. To quantify the diversity of lyrics, we compute the compression rate^36^, effectively capturing the repetitiveness of lyrics and several diversity measures. For structural descriptors of lyrics, we utilize the descriptors identified by Malheiro et al.^37^, which, for instance, include the number of times the chorus is repeated, the number of verses and choruses, and the alternation pattern of verse and chorus. For descriptors that capture rhymes contained in lyrics, we extract, for instance, the number of subsequent pairs of rhyming lines (i. e., clerihews), alternating rhymes, nested rhymes, or alliterations, but also rely on general descriptors such as the fraction of rhyming lyrics^30^ as these have been shown to be characteristic for the style of lyrics^30^. For measuring readability, we use standard measures like the number of difficult words, or Flesch’s Reading Ease formula^38^. Furthermore, we extract emotional descriptors from lyrics by applying the widely used Linguistic Inquiry and Word Count (LIWC) dictionary^39^, which has also been applied to lyrics analyses^18,37,40^. We provide a complete list of all descriptors, including a short description and further information about how the descriptors are extracted in Table 1.

**Table 1:** List of all lyrical descriptors extracted for the two datasets, including a brief description.

Name	Description
Lexical descriptors	
Line counts	Total number of lines, blank lines, unique lines, ratio of blank and repeated lines
Token counts	Number of tokens, characters, repeated token ratio, unique tokens per line, and avg. tokens per line
Character counts	Number of [!?.,:;”-()] and digits (total amount of these characters and individual counts per character), ratio of punctuation and digits
Token length	Average length of tokens
n-gram ratios	Ratio of unique bigrams and trigrams
Legomenon ratios	Ratio of hapax legomena, dis legomena and tris legomena
Parts of speech	Frequency of adjectives, adverbs, nouns, pronouns, verbs
Past tense	Percentage of verbs in past tense
Stop words	Number and ratio of stop words, stop words per line
Uncommon words	Number of uncommon words (i.e., words not contained WordNet^60^)
Diversity descriptors	
Compression ratio	Ratio of the size of *zlib* compressed lyrics vs. the original, uncompressed lyrics
Diversity measures	Measure of Textual Lexical Diversity (MTLD), Herdan’s *C*, Summer’s *S*, Dugast’s $U^2$ and Maas’ $a^2$
	The diversity descriptors were extracted using the Python lexical_diversity and lexicalrichness library.
Readability descriptors	
Readability formulas	Flesch Reading Ease, Flesch Kincaid Grade, SMOG (Simple Measure of Gobbledygook), Automated Readability Index, Coleman Liau Index, Dale Chall Readability Score, Linsear Write Formula, Gunning Fog, Fernandez Huerta, Szigriszt Pazos and Gutierrez Polini
Difficult words	Number of difficult words (consisting of three or more syllables)
	The readability descriptors were extracted using the Python textstat library.
Rhyme descriptors	
Rhyme structures	Numbers of couplets, clerihews, alternating rhymes and nested rhymes
Rhyme words	Number of unique rhyming words, percentage of rhyming lines in the lyrics
Alliterations	Number of alliterations of length two, three, and four or more
	The rhyme descriptors were extracted using the Python pronouncing library, which provides an interface to the Carnegie Mellon University Pronouncing Dictionary.
Structural descriptors	
Element counts	Number of sections and verses
Distribution	Relation between the number of verses vs. sections and number of choruses vs sections
Title occurrences	Number of times the song’s title appears
Pattern	Verse and chorus alternating, two verses and at least one chorus, two choruses and at least one verse
Start	Starts with chorus (binary attribute)
Ending	Ends with two chorus repetitions (binary attribute)
Emotional descriptors	
Sentiment scores	Positivity and negativity scores via AFINN^61^, the sentiment lexicon by Bing Liu et al.^62^, the MPQA opinion corpus^63^, the sentiment140 dataset^64^ and the SentiWordNetlexicon^65^
NRC	Emotion scores according to the NRC affect intensity lexicon^66^
LIWC	Descriptors provided by LIWC^39^
Happiness	Happiness score according to labMT^67^

### Statistical analyses

Figure 1 provides an overview of the methodological framework used for the analyses presented. The two analyses conducted aim to (1)  investigate the evolution of descriptors over five decades by performing a release year regression task to identify the importance of descriptors, and (2)  investigate the interplay of lyrics descriptors, release year, and lyrics view count by performing a regression analysis on a dataset containing 12,000 songs, balanced for both genres and release years. The combination of these two analyses provides us with complementary findings; while the first analysis uses the entirety of our collected dataset and therefore derives general findings on descriptor importance, the second analysis, performed on a carefully balanced, reduced dataset, provides us with a more in-depth analysis on the strength of relationships of the individual lyrics and popularity descriptors and temporal aspects.

#### Analysis 1: evolution of descriptors and descriptor importance

In this analysis, we investigate which descriptors are most strongly correlated with the release year of a song. We expect that descriptors that correlate more strongly with the release year lead to better-performing regression models. Therefore, we train a release year regressor for each of the five genres. We are mostly interested in determining each descriptor’s importance, thereby identifying the descriptors that are most effective at predicting a linear trend across release years.

First, we perform z-score normalization of the descriptors. Subsequently, we remove multicollinear descriptors using the variance inflation factor (VIF). Here, we iteratively remove descriptors that exhibited a VIF higher than 5 until all of the remaining descriptors have a VIF lower than 5 (as also performed in Analysis 2). Preliminary analyses showed that the data associated with individual descriptors are heteroscedastic (i.e., the variance is not homogenous, but dependent on the release year). To overcome this bias, we use Huber’s M regressor^41^, a widely used robust linear regressor.

**Figure 2:**
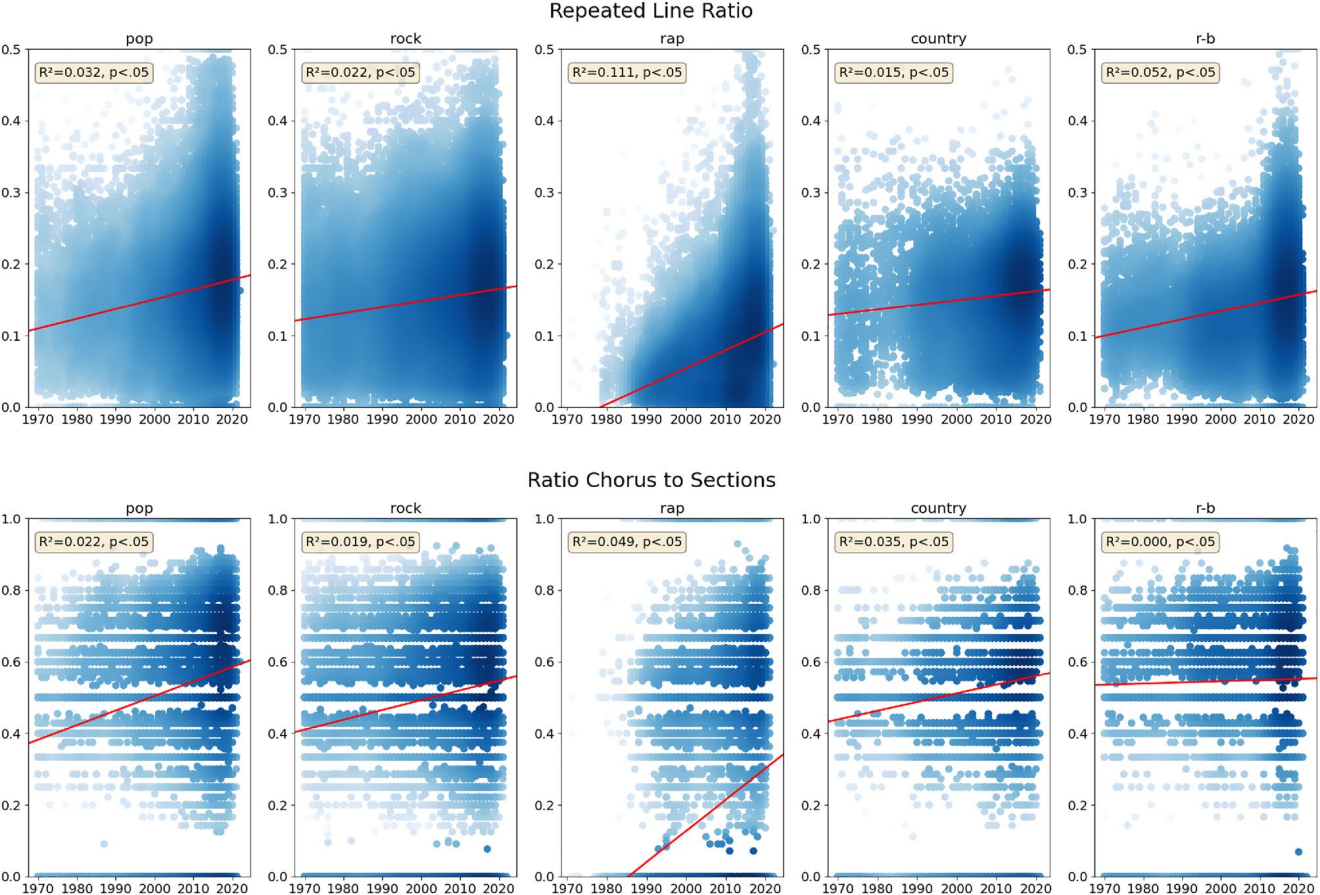
Evolution over time for the descriptors *repeated line ratio* and *ratio chorus to sections* for each genre. The linear regression lines (in red) show the evolution of descriptor values over time for each descriptor and genre (Huber’s M robust regression models are trained individually for each descriptor and genre combination). Blue colors denote the density of data points in a given region. R^2^ and p-values are provided in the yellow boxes.

Notably, we perform these analyses on all available songs for the five genres for which we can successfully extract all descriptors (totaling to 353,320 songs). For each genre, we train the regression model and analyze the model’s performance and the computed regression coefficients to identify the most important descriptors for determining the release year of the songs. The models for this analysis are built in Python, using the statsmodels package^42^ (via the Robust Linear Models (RLM) class; statsmodels version 0.14). The plots in Fig. 2 are generated using the Matplotlib library^43^ (version 3.7.0) and used gaussian_kde of scipy.stats for the density computation^44^.

#### Analysis 2: interplay of lyrics descriptors, lyrics view count, and release year

In this analysis, we first assess whether lyrics’ view count is related to the underlying musical genre and to which extent such a connection might vary over time. Subsequently, we evaluate the evolution of pop music lyrics over time within each musical genre. We assume that lyric’s view count varies amongst musical genres, and these variations can be related to changes in the lyrics over time. Thus, to further deepen our understanding of the relationship between lyrics’ view count and genre, as well as whether the release date has a role in this relationship, we start by performing a multinomial logistic regression analysis considering genre as the dependent variable and the interaction between popularity and release year as predictors, where the number of views of the lyrics of a song is used to capture lyrics popularity. Subsequently, since the lyrics from different musical genres might develop differently over time, to investigate the relationship between release date and lyrics descriptors, a different linear model (considering release year as a dependent variable) is fitted for each genre individually. To find the model that best fits the data for each genre, we apply the backward stepwise method as appropriate in our exploratory study. From the stepwise methods, we consider backward elimination over forward selection to minimize the risk of excluding predictors involved in suppressor effects^45^.

To carry out a fair comparison, before starting the analyses, the collected dataset is randomly downsampled to guarantee a balanced distribution of songs across musical genres and years. To enable this, due to the highly skewed distribution of data over time, only the last three decades (1990–2020) could be considered for this analysis. The means and standard deviations of both the whole and the downsampled datasets are mostly comparable across the musical genres. There is, however, a larger difference between the standard deviations for pop and country. Unstandardized means and standard deviations for the whole dataset vs. the downsampled for both the lyrics views and the play count are shown in Table 2.

**Table 2:** Unstandardized means and standard deviations ($\mu \pm \sigma$) for the whole dataset (All) versus the downsampled (Down).

Genre					
	Rap	Pop	Rock	R&B	Country
Play count					
All	$147 \pm 485$	$358 \pm 1,214$	$796 \pm 2,218$	$267 \pm 900$	$330 \pm 785$
Down	$140 \pm 375$	$419 \pm 1,367$	$796 \pm 2,127$	$275 \pm 1,024$	$356 \pm 771$
Lyrics view					
All	$48,377 \pm 251,109$	$18,640 \pm 142,764$	$9,741 \pm 53,505$	$35,821 \pm 183,550$	$6,920 \pm 40,176$
Down	$41,787 \pm 202,711$	$12,021 \pm 67,331$	$11,516 \pm 55,599$	$28,067 \pm 212,270$	$5,774 \pm 28,046$

**Figure 3:**
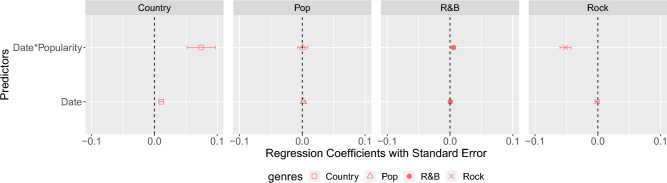
Forest plot displaying the estimated multinomial logistic regression coefficients (standardized beta) for the prediction of musical genre. As reference class, *rap* i. e., the genre with the highest average lyrics view count, is considered.

A total of 2400 items, i. e., songs, are considered for each musical genre. Due to the high diversity across the measurement unit of the predictors, i. e., popularity scores and lyrics descriptors, these are z-score normalized and multicollinear outliers are identified by computing Mahalanobis distance^46^ and subsequently removed. Highly correlated descriptors are also discarded until all of them presented a variance inflation factor less than 5. The results from the multinomial logistic regression show that lyrics view count differs across decades for the evaluated genres. Therefore, we investigate the relation between lyrics view count and particular lyrics descriptors by also fitting a multiple linear regression model containing the interaction between the lyrics view count and the other predictors. However, the model with the interaction is not significantly better than the baseline model (for all the musical genres, analysis of variance yields $p>.01$); thus, only the model without interaction is considered in the evaluation of the multiple linear regression results for each genre. The statistical models of Analysis 2 are built on the statistical software R^47^ version 4.1.2 (2021-11-01). Multinomial logistic regression is carried out using the mlogit package^48^ (version 1.1-1) while the linear models for each genre are fitted with the nlme package^49^ (version 3.1-155) and multiple comparisons across genres are performed with the multcomp package^50^ (version 1.4-25). The graphic shown in Fig. 3 is generated with the ggplot2 package^51^ (version 3.4.3).

## Results and discussion

### Analysis 1: evolution of lyrics and descriptor importance

In this analysis, we are particularly interested in the most important and hence, most characteristic features for the task of per-genre release year regression. The top ten descriptors (i.e., the descriptors with the highest regression coefficients) for each of the five genres in our dataset are given in Table 3. The R^2^ values obtained per genre are 0.0835 for pop, 0.0699 for rock, 0.3340 for rap, 0.2510 for R&B, and 0.1267 for country.

**Table 3:** Lyric descriptors identified by robust regression (Huber M regressor).

Genre					
	Pop	Rock	Rap	Country	R&B
1	Unique rhyme words^Rh^	Repeated line ratio^L^	Ratio verses to sections^S^	Punctuation ratio^L^	Repeated line ratio^L^
2	Repeated line ratio^L^	Title occurrences^S^	Unique rhyme words^Rh^	Compression ratio^D^	Dugast’s U^D^
3	Ratio chorus to sections^S^	Number of verses^S^	Ratio chorus to sections^S^	Blank line count^L^	Unique rhyme words^Rh^
4	Title occurrences^S^	Avg. token length^L^	Repeated line ratio^L^	Tris legomenon ratio^L^	Blank line count^L^
5	Ratio verses to sections^S^	Dot count^L^	Dot count^L^	Avg. token length^L^	Parenthesis count^L^
6	Dot count^L^	Ratio chorus to sections^S^	Hyphen count^L^	Positive emotion^E^	Positive emotion^E^
7	Dugast’s U^D^	Unique rhyme words^Rh^	Exclamation mark count^L^	Blank line ratio^L^	Rhyme percent^Rh^
8	Avg. token length^L^	Dale-Chall readability score^R^	MTLD^D^	Unique rhyme words^Rh^	MTLD^D^
9	Parenthesis count^L^	Dis legomenon ratio^L^	Dugast’s U^D^	Number of couplets^Rh^	Dot count^L^
10	Pronoun frequency^L^	Punctuation Ratio^L^	Blank line count^L^	Ratio chorus to sections^S^	Anger^E^

Superscripts denote descriptor categories, with L = lexical, E = emotion, S = structural, Rh = rhyme, Re = readability, and D = diversity.

We can identify several descriptors that are among the most important for multiple genres. The number of *unique rhyme words* is among the top ten descriptors across all five genres analyzed. The *number of dots* and *repeated line ratio* descriptors are among the top descriptors in pop, rock, rap, and R&B. Four descriptors are featured in the top descriptors of three genres each (*ratio verses to sections*, *ratio choruses to sections*, *average token length*, *Dugast’s U* (modelling the number of token types as a function of the token count), and *blank line* count. Interestingly, when considering a higher abstraction level (i.e., descriptor categories such as lexical, emotion, structure, rhyme, readability, or diversity), we observe that lexical descriptors emerge as the predominant category across all five genres. Furthermore, at least one rhyme descriptor is also among the top descriptors for each genre. Four out of the five genres also feature at least one structural descriptor, while R&B does not. While for pop and rap, the top-10 descriptors contain lexical, structural, diversity, and rhyme descriptors, rock features a readability descriptor. For country, five categories of descriptors are within the top 10. Interestingly, descriptors measuring the lexical diversity of lyrics are among the top descriptors for rap and R&B (*Dugast’s U*; and Measure of Textual Lexical Diversity *MTLD* that captures the average length of sequential token strings that fulfil a type-token-ratio threshold), country (*compression ratio*, i.e., ratio of the size of zlib compressed lyrics compared to the original, uncompressed lyrics), and pop (*Dugast’s U*). Emotion descriptors only occur among the most important descriptors for country (*positive emotion*) and R&B (*positive emotions and anger*). Readability descriptors are among the top descriptors for rock (*Dale-Chall* readability score, which is computed based on a list of 3000 words that fourth-graders should be familiar with).

Figure 2 shows the distribution of descriptor values for *repeated line ratio* and *ratio of choruses to sections* over time, separately for each of the five genres. Each genre is analyzed separately, with a robust regression model trained for each descriptor-genre combination; the resulting regression lines are depicted in red. The *repeated line ratio* increases over time for all five genres, indicating that lyrics are becoming more repetitive. This further substantiates previous findings that lyrics are increasingly becoming simpler^11^ and that more repetitive music is perceived as more fluent and may drive market success^52^. The strongest such increase can be observed for *rap* (slope $m = 0.002516$), whereas the weakest increase is displayed by *country* ($m = 0.000640$). The *ratio of chorus to sections* descriptor behaves similarly across different genres. The values for this descriptor have increased for all five genres. This implies that the structure of lyrics is shifting towards containing more choruses than in the past, in turn contributing to higher repetitiveness of lyrics. We see the strongest growth in the values of this descriptor for *rap* ($m = 0.008703$) and the weakest growth for *R&B* ($m = 0.000325$). The fact that the compression ratio descriptor (not shown in the figure) also shows an increase in all genres except R&B further substantiates the trend toward more repetitive lyrics. Another observation is that the lyrics seem to become more personal overall. The *pronoun frequency* is increasing for all genres except one (*country* with $m = -0.000145$). The strongest increase can be observed for *rap* ($m = 0.000926$), followed by *pop* ($m = 0.000831$), while *rock* ($m = 0.000372$) and *R&B* ($m = 0.000369$) show a moderate increase. Furthermore, our analysis shows that lyrics have become angrier across all genres, with *rap* showing the most profound increase in *anger* ($m = 0.015996$). Similarly, the amount of *negative emotions* conveyed also increases across all genres. Again *rap* shows the highest increase ($m = 0.021701$), followed by *R&B* ($m = 0.018663$), while *country* shows the lowest increase ($m = 0.000606$). At the same time, we witness a decrease in *positive emotions* for *pop* ($m = -0.020041$), *rock* ($m = -0.012124$), *country* ($m = -0.021662$), and *R&B* ($m = -0.048552$), while *rap* shows a moderate increase ($m = 0.000129$).

### Analysis 2: interplay of lyrics descriptors, view counts, and release year

The second set of analyses first aims at investigating the interplay between lyrics descriptors, release year, and listening as well as lyrics view count. The employed multinomial logistic regression fits significantly better the data than the baseline model, i. e., a null model without predictors, indicating an increase in the explained variability (likelihood ratio chi-square of 314.56 with a $p<.0001$ and McFadden pseudo R^2^ of 0.01).

To assess the effect of the predictors, the genre class rap (i. e., the one with the highest average lyrics view count), is considered as the reference class of the dependent variable. Our results show that the probability of a song being from country or rock instead of rap, according to its *lyrics view count*, varies across decades. As *lyrics view count* increases, the effect of the year slightly augments (in 1.07 odds) the probability of a song being from country instead of from rap: $\beta (SE)=0.07(0.02)$, $z=3.29$, $p=.0009$. Differently, as *lyrics view count* increases, the effect of a raising year decreases (in 0.94 odds) the probability of a song being from rock instead of from rap: $\beta (SE)=-0.05(0.01)$, $z=-5.89$, $p<.0001$. In other words, the lyrics of older rock songs are generally more popular than new ones in comparison to rap, and vice versa for country. This is visualized in Fig. 3, showing the estimated effects of the multinomial logistic regression model. The interaction between *lyrics view count* and *release year* did not show a significant effect for rap with respect to pop and R&B (cf. date*popularity for pop and R&B). Differently, for country and rock, the estimated regression coefficients are positive and negative, respectively (cf. date*popularity for country and rock). This shows that compared to rap, lyrics’ popularity increases over time for country, while decreasing for rock. The same analysis is performed considering *song listening count* instead of lyrics view count, i. e., we perform multinomial logistic regression considering *genre* as the dependent variable and the interaction between *listening count* and *release date* as predictors. This analysis shows that the *release date* does not affect the relationship between *listening count* and *genre*, as no significant effects are shown. While track listening counts do not show any effects, lyrics view counts do indeed show effects; suggesting that for some musical genres, fans’ interest in lyrics goes beyond their listening consumption. In other words, while the play counts for a given genre might not significantly differ, when it comes to the listeners’ interest in lyrics, clear patterns are displayed: rock fans mostly enjoy lyrics from older songs; country fans are more interested in new songs’ lyrics. However, the small determination coefficient of the multinomial logistic regression shows that the explanatory power of the model is limited; thus, documented significant p-values might partially result, due to the huge sample size, from random noise.

In Table 4, the results from the individual multiple linear regression performed for each genre are given. We find that for rap, the most variance of the release year (the dependent variable) can be explained by the predictors. $32\%$ (i. e., $R^{2}=0.32$) of the variance in the release year for rap can be explained by the descriptors extracted from the lyrics. This is not surprising as rap, characterized by the use of semi-spoken rhymes, is a musical style that has grown in the context of practices marked by high-level linguistic competencies, such as competitive verbal games^53^. Indeed, among the evaluated musical genres, rap is the one in which lyrics play the most prominent role. The second genre for which a higher amount of variance in the release year is explained by the descriptors extracted from the lyrics is R&B ($R^{2}=0.20$). This might be explained, from a musicological perspective, by taking into account the relationship between R&B and other musical genres. For instance, R&B was simplified concerning the lyrics (besides the music) by eliminating adult-related themes and topics^54^. As such, it was a precursor to the development of rock-and-roll, which explains the higher importance of lyrics in modeling the evolution of R&B with respect to rock. Note that although we utilize R&B as a general musical genre, other terms subsequently introduced relating to R&B, such as *soul*, are also considered under the umbrella of R&B. This is particularly important as we investigate music released in more recent decades (1990–2020) with respect to the time when the term R&B was originally coined. At the same time, it highlights the importance of historically contextualizing the musical genres assessed in the comparison, since beyond their intrinsic characteristics (e. g., lyrics having a central role in rap), also their heterogeneous nature, in this case, R&B being more heterogeneous than rock e.g., blues and funk being highly repetitive, while soul has undergone substantial changes and is now lyrically in pop song form), might also have an impact in the role played by lyrics. For pop, rock, and country, the amount of variance in release date explained by the predictors is lower than for rap and R&B, with an $R^{2}$ value of 0.09 for pop and rock, 0.11 for country. This indicates that, unlike rap, and to some extent R&B, lyrics might not be a very meaningful indicator of the development of other musical genres.

**Table 4:** Results of the best-fitting models for the predictors for predicting the release year, considering each genre individually (rap, pop, rock, R&B, and country). $\beta$ coefficient, standard error (*SE*), and *p*-value are reported.

	Rap			Pop			Rock			R&B			Country		
	$\beta$	*SE*	*p*	$\beta$	*SE*	*p*	$\beta$	*SE*	*p*	$\beta$	*SE*	*p*	$\beta$	*SE*	*p*
Lyrics view count	0.22	0.10	.031				$-1.47$	0.42	${\textbf {.000}}$	0.32	0.11	${\textbf {.003}}$			
Complexity															
Compression ratio	0.70	0.38	${\text {.065}}$							$-0.96$	0.26	${\textbf {.000}}$			
Readability															
SMOG	−0.57	0.13	${\textbf {.000}}$				0.69	0.28	.013	0.32	0.201	0.116	0.98	0.30	${\textbf {.001}}$
Difficult words				−0.77	0.32	.016	−1.37	0.39	${\textbf {.000}}$				−1.36	0.51	${\textbf {.007}}$
Dale-Chall				0.55	0.21	**.009**	1.18	0.36	${\textbf {.001}}$						
Coleman-Liau										0.75	0.22	**0.001**			
Lexical															
Blank line count				0.35	0.17	.041	0.86	0.31	**.005**	$-0.55$	0.19	${\textbf {.003}}$	1.38	0.38	${\textbf {.000}}$
Blank line ratio	$-0.70$	0.19	${\textbf {.000}}$				$-0.49$	0.26	.060				$-1.40$	0.28	${\textbf {.000}}$
Repeated line ratio	2.59	0.29	${\textbf {.000}}$	1.35	0.19	${\textbf {.000}}$	1.10	0.22	${\textbf {.000}}$	3.19	0.24	${\textbf {.000}}$	0.51	0.21	${\text {.016}}$
Exclamation mark count	$-0.32$	0.10	${\textbf {.001}}$	$-0.34$	0.19	.076							$-1.65$	0.70	${\text {.018}}$
Question mark count	$-0.24$	0.13	${\text{.075}}$				0.93	0.30	**.002**						
Hyphen count	$-0.84$	0.17	${\textbf {.000}}$				0.69	0.25	${\textbf {.005}}$				$-0.78$	0.38	.039
Comma count	0.29	0.17	.091	1.37	0.24	${\textbf {.000}}$	1.36	0.47	${\textbf {.003}}$	0.55	0.22	0.015			
Parens count	0.68	0.15	${\textbf {.000}}$	$-0.58$	0.22	${\textbf {.009}}$	0.98	0.44	${\text {.025}}$	$-0.52$	0.15	${\textbf {.000}}$			
Punctuation ratio	$-0.58$	0.29	.047				$-1.28$	0.38	${\textbf {.000}}$				2.04	0.35	${\textbf {.000}}$
Stop words ratio	$-0.53$	0.28	.059				0.56	0.22	.011	$-0.74$	0.27	${\textbf {.005}}$			
Stop words per line										1.20	0.35	${\textbf {.000}}$			
Dis legomenon ratio	$-0.98$	0.28	${\textbf {.000}}$				$-0.62$	0.18	${\textbf {.001}}$	$-0.75$	0.21	**.001**			
Tris legomenon ratio	0.61	0.33	.063										0.81	0.17	${\textbf {.000}}$
Maas	$-1.17$	0.38	${\textbf {.002}}$							$-1.11$	0.26	${\textbf {.000}}$			
Pronoun frequency	2.25	0.27	${\textbf {.000}}$	0.45	0.16	${\textbf {.005}}$				0.71	0.22	${\textbf {.001}}$	$-0.66$	0.19	${\textbf {.001}}$
Past tense ratio							$-0.24$	0.17	.148	0.37	0.19	.054	$-0.57$	0.15	${\textbf {.000}}$
Structure															
Title occurrences							1.34	0.25	${\textbf {.000}}$						
Number of sections	$-0.62$	0.25	.016	$-0.37$	0.25	.136	$-0.79$	0.23	${\textbf {.001}}$	$-0.72$	0.27	${\textbf {.008}}$			
Ratio verses sections	1.46	0.20	${\textbf {.000}}$	0.59	0.23	.012							$-0.85$	0.25	${\textbf {.001}}$
Ratio chorus sections	1.11	0.29	${\textbf {.000}}$	0.95	0.28	${\textbf {.001}}$	0.82	0.24	${\textbf {.000}}$	0.65	0.33	.047			
Alternation verse chorus							$-0.46$	0.16	${\textbf {.005}}$	0.52	0.21	.012	$-0.25$	0.14	.065
Two verses/one chorus	0.73	0.22	${\textbf {.001}}$							$-0.77$	0.26	${\textbf {.003}}$			
Rhyme															
Number alternating	$-0.58$	0.16	${\textbf {.000}}$										$-0.85$	0.46	.066
Number nested				$-0.24$	0.17	.150	$-0.60$	0.19	${\textbf {.002}}$				1.19	0.42	${\textbf {.005}}$
Rhyme percent	1.20	0.29	${\textbf {.000}}$							0.68	0.21	${\textbf {.001}}$			
Unique rhyme words	$-1.91$	0.16	${\textbf {.000}}$	$-0.94$	0.22	${\textbf {.000}}$	$-1.40$	0.24	${\textbf {.000}}$	$-1.54$	0.26	${\textbf {.000}}$	$-0.92$	0.23	${\textbf {.000}}$
Alliterations length 4				0.23	0.14	.113	$-0.64$	0.22	${\textbf {.004}}$	0.26	0.14	.070			
Emotion (LIWC)															
Anger	0.51	0.14	${\textbf {.000}}$	0.26	0.16	.111				1.91	0.30	${\textbf {.000}}$	0.77	0.32	.016
Sad	0.95	0.30	${\textbf {.001}}$										$-0.39$	0.16	.015
Positive emotions	0.62	0.27	${\text {.024}}$	$-0.84$	0.15	${\textbf {.000}}$				$-0.89$	0.14	${\textbf {.000}}$	$-0.71$	0.18	${\textbf {.000}}$

Significance below the threshold of .01 is highlighted. Due to space constraints, we only report fixed effect estimates showing meaningful impact (i. e., $p<.01$) for at least one genre. Adjusted R^2^ for each genre is: rap (0.32), pop (0.09), rock (0.09), R&B (0.19), and country (0.11).

The results show that lyrics’ view count has a relevant effect in predicting the release years of songs only for R&B and rock music. For R&B, there is a positive relationship between *release year* and *lyrics view count*: $\beta = 0.32$, $p=.003$; cf. *lyrics view count* for R&B in Table 4. This indicates that new songs are more popular than old ones in terms of lyrics views. Differently, for rock, as expected from the outcomes obtained in the multinomial logistic regression, a strong negative relationship between *release year* and *lyrics view* is shown: $\beta = -1.47$, $p<.000$; cf. *lyrics view count* for rock in Table 4. This indicates that old rock songs are more popular than recent ones, which can be interpreted from a sociological perspective. Unlike pop, which can be seen as more “commercial” and “ephemeral”, targeting a young audience and whose value is typically measured by record sales, rock has commonly targeted a middle-class audience more interested in tradition and often (ideologically) defeating commercialism^55^.

Properties of the lyrics related to complexity and readability, i. e., indicators of the repetitiveness and the difficulty associated with the understanding of a written text, respectively, seem to exhibit meaningful changes over time for rap, and to a lesser extent for pop, rock, and R&B. Confirming previous work^11^, the complexity and difficulty of the lyrics seem to decrease with time for some musical genres. Concerning complexity, this is displayed by the positive $\beta$ for *compression ratio* (essentially capturing the repeatability of lyrics) shown by rap (cf. $\beta = 0.70$ in Table 4). This indicates that rap lyrics become easier to comprehend over time, something that can be interpreted as a sign of increasing repetitiveness and, therefore, simplicity. However, the opposite trend is shown for R&B (cf. $\beta = -0.96$, in Table 4), which suggests that the simplification over time might depend on the musical genre; indeed, this descriptor is not relevant neither for pop nor rock nor country. The decline in lyrics’ difficulty observed over time for rap is confirmed by the negative $\beta$ for *Simple Measure of Gobbledygook* (SMOG) readability measure (in a sample of 30 sentences, words with three or more syllables are counted and used to compute the final SMOG score). This indicates a detriment in complexity concerning the lyric’s readability (cf. $\beta = -0.57$ in Table 4). The increase in readability over time is also confirmed for rock, as shown by the positive slope for  *Dale-Chall* readability score (cf. $\beta = 1.18$ in Table 4).

As expected, the results also show that lexical descriptors have a more prominent role in rap, i.e., the musical genre for which lyrics are most relevant. Indeed, when calculating the predictors block-wise across feature types, this is the type of feature showing the highest adjusted R^2^: for rap (0.22), followed by R&B (0.13). Block-wise adjusted R^2^ per genre for each feature type are as follows. For rap: Complexity (0.04), Readability (0.04), Lexical (0.22), Structure (0.10), Rhyme (0.13), Emotion (0.02); for pop: Readability (0.01), Lexical (0.06), Structure (0.02), Rhyme (0.01), Emotion (0.01); for rock: Readability (0.01), Lexical (0.04), Structure (0.03), Rhyme (0.01); for R&B: Complexity (0.01), Readability (0.01), Lexical (0.13), Structure (0.02), Rhyme (0.02), Emotion (0.04); for country: Readability (0.014), Lexical (0.09), Structure (0.02), Rhyme (0.02), Emotion (0.01). *Repeated line ratio* is the only descriptor showing a meaningful impact for all the genres, confirming the results of Analysis 1. The relationship between this descriptor and the release year is positive for all of them (cf. positive $\beta$ in Table 4), which indicates that lyrics become more repetitive over time in all the evaluated genres. This trend is confirmed by the negative relationship between release year and the *Maas* score, a measure for lexical diversity proposed by H.-D. Maas^56^ (the score models the type-token ratio (i.e., the ratio of the total number of words and the total number of unique terms) on a log scale), shown for all the genres except country, pop, and rock (for which this descriptor is not included in the model as it did not show a significant contribution), which indicates that vocabulary richness decreases with time (cf. negative $\beta$ for *Maas* in Table 4). As already mentioned, step-wise backward elimination is used to find the best-fitting model for each musical genre. The trend toward simplicity over time can also be observed in the structure, which shows a decrease in the *number of sections*, most prominently shown for R&B and rock (cf. $\beta = -0.72$ and $\beta = -0.79$, respectively in Table 4); as well as a general increment (except for country) in the ratio between verses to chorus and verses to sections (cf. positive $\beta$ for *ratio verses to sections* and *ratio chorus to sections* in Table 4). Similarly, the results for rhyme-related descriptors further confirm the tendency towards simpler lyrics over time for all musical genres. This is particularly shown by the increment of the *rhyme percent* in rap in R&B (cf. $\beta = 1.20$ and$\beta = 0.68$, respectively) and by a detriment in the number of rhyme words (cf. negative $\beta$ for all the genres), which shows a decline in the rhymes’ variety over time. However, for block-wise predictors, slightly higher adjusted R^2^ for structure and rhyme are only shown for rap (0.10 and 0.13, respectively), but not for the other musical genres.

Concerning emotion descriptors, the musical genre in which these play the most important role is rap, followed by R&B. For R&B the results show that the content of the lyrics becomes more negative with time, as shown by the increase in concepts related to *anger* and a detriment in *positive emotions* (cf. $\beta = 1.91$ and $\beta =-0.89$, respectively, in Table 4). Differently, for rap, there is a general increase in the use of emotion-related words with time, both negative and positive (cf. positive $\beta$ for all the emotion descriptors), which indicates a tendency towards the use of more emotional words. Confirming outcomes from previous work^13^, as shown for R&B, also for pop and country, a tendency toward more negative lyrics is displayed over time; for rock, emotion seems to play a negligible role in the evolution of lyrics. As a final note, we would like to emphasize that since both the overall and block-wise adjusted R^2^ are very low, these results should be interpreted cautiously, and taken as tendencies rather than strong differences and could partly result from partly non-randomness in subsampling.

### Result summary for both studies

Regarding RQ1 (Which trends can we observe when correlating multifaceted lyrics descriptors with temporal aspects in an evolution analysis?), we come to the following conclusion: Despite minor contradictory outcomes concerning complexity and readability for rap and R&B, the interpretation of the lyric’s lexical component, structure, and rhyme, for all investigated genres, generally shows that lyrics are becoming simpler over time^11^, as shown both analyses. This is shown by a decline in vocabulary richness for some specific genres, i.e., rap and R&B, and by a general increase in repetitiveness for all the evaluated musical styles. Besides this, lyrics seem to become more emotional with time for rap, and less positive for R&B, pop, and country. Also, we observe a trend towards angrier lyrics across all genres except for rock. Potential reasons for the trend towards simpler lyrics are discussed by Varnum et al.^11^. They speculate that this might also be related to how music is consumed, technological innovation, or the fact that music is mostly listened to in the background. As for RQ2 (Which role does the popularity of songs and lyrics play in this scenario?), we conclude that while song listening counts do not show any effects, lyrics view counts do indeed show effects. This suggests that for rap, rock, and country, lyrics play a more pronounced role than for other genres and that listeners’ interest in lyrics goes beyond musical consumption itself.

### Limitations

While our analyses resulted in interesting insights, they have certain limitations, which we would like to discuss in the following. Most of these relate to the various challenges pertaining to data acquisition, and the resulting biases in the data we investigated.

The two main data sources for our investigation are last.fm and Genius. Given the nature and history of these platforms, in particular last.fm, the studied LFM-2b dataset is affected by community bias and popularity bias. As for *community bias*, while last.fm does not release official statistics of their users, research studies conducted on large amounts of publicly available demographic and listening data have shown that the last.fm’s user base is not representative of the global (or even Western) population of music listeners^28,29^. In particular, the last.fm community represents music aficionados who rely on music streaming for everyday music consumption. They are predominantly male and between 20 and 30 years old. The community is also strongly dominated by users from the US, Russia, Germany, and the UK^29^, whose music taste does not generalize to the population at large^57^. The findings of our analyses, in particular Analysis 2, which investigates user-generated music consumption data, are therefore valid only for this particular subset of music listeners. Also directly related to our data sources, and, particularly, the Genius platform is the genre information used in our analyses. Annotators and editors on the Genius platform may assign one of six high-level genres and an arbitrary number of so-called secondary tags (i.e., subgenres) to each song. The alignment of genre and subgenre assignments is quality-checked by the community, no genre hierarchy is used to check the validity of the genre and subgenre assignments, which can introduce malformed genre assignments.

In addition, the last.fm data on listening counts and the Genius data on lyrics view counts are prone to *popularity bias*. More precisely, these counts for songs released before the emergence of the platforms (2002 and 2009, respectively, for last.fm and Genius) underestimate the true frequencies of listening and lyrics viewing. On the one hand, this is due to the platforms’ demographic structure of users (see above); but also because a majority of vinyl and cassette (and even some CD) releases have never found their way into these digital online platforms. This kind of popularity bias in our investigated data might have significantly influenced the trends identified for the 1970s to 2000s. However, only Analysis 2 might have been affected by this since popularity estimates are not used in Analysis 1. And even for Analysis 2, popularity values are Z-score normalized, which to some extent accounts for this kind of bias. Still, it should also be pointed out that the randomization strategy might have led to a sampling bias in terms of popularity. This might have partly affected the results, eventually introducing a bias for the genres pop and country, which due to the mentioned limitation, should be taken particularly cautiously.

Both limitations, related to demographic bias and popularity bias, could be overcome by resorting to other data sources, notably the often-used Billboard Charts. However, using this data would introduce other distortions, among others, a highly US-centric view of the world, a much more limited sample size, and a lower granularity of the popularity figures (only ranks instead of play counts). In addition, Billboard Charts are only indicative of music consumption, not for lyrics viewing, which we particularly study in this paper.

Another limitation of the work at hand is the *restriction to English lyrics*. This choice had to be made to ensure a language-coherent sample of songs and, consequently, the comparability of results. While some of the descriptors could have been computed for other languages as well, due to the different characteristics of languages (e.g., different lexical structures), a cross-language comparison of the descriptors would not be meaningful. Also, most of the resources required to compute the readability scores and emotional descriptors are only available for English. Here we note that for assessing the readability of texts, we rely on rather simple metrics. For instance, difficult words are defined as words with three or more syllables. We also note that some of the readability metrics rely on sentences, which might not always be directly extractable from lyrics. Nevertheless, in future work, we could include more languages and conduct analyses on songs within each language class on a limited set of suited descriptors.

Furthermore, we also acknowledge the changing record distribution landscape, a further limitation of this work. These changes are shown, for instance, by the IFPI’s Global Music Report 2023^58^, provides evidence of the decline of physical sales revenue vs. the steady increase in streaming revenue in the last two decades. This not only changed, for instance, the number of songs on an album as this was physically restricted on vinyl or CDs, but also the way songs are sequenced^59^. On streaming platforms, a song is considered consumed if it is played for at least 30 seconds. Hence, artists aim to start their songs with easily identifiable melodies and lyrics.

Regarding the models employed, we note that these models assume that the changes in individual features across the analyzed genres are linear. While the change of some of the descriptors has been shown to be linear (for instance, lyrics simplicity (compressibility)^11^ or brightness, happiness, or danceability^23^), this might not be the case for all of the descriptors we employ in our studies. In fact, for instance, concreteness has been shown to decline until the 1990s and then increase^9^.

## Conclusion

Our study examines the evolution of song lyrics over five decades and across five genres. From a dataset of 353,320 songs, we extracted lexical, linguistic, structural, rhyme, emotion, and complexity descriptors and conducted two complementary analyses. In essence, we find that lyrics have become simpler over time regarding multiple aspects of lyrics: vocabulary richness, readability, complexity, and the number of repeated lines. Our results also confirm previous research that found that lyrics have become more negative on the one hand, and more personal on the other. In addition, our experimental outcomes show that listeners’ interest in lyrics varies across musical genres and is related to the songs’ release year. Notably, rock listeners enjoy lyrics from older songs, while country fans prefer lyrics from new songs.

We believe that the role of lyrics has been understudied and that our results can be used to further study and monitor cultural artifacts and shifts in society. For instance, we could combine and compare the studies on the changing sentiment in societies and shifts in the use of emotionally loaded words and the sentiment expressed in the lyrics consumed by the different audiences (age, gender, country/state/region, educational background, economical status, etc.). From a computational perspective, establishing a deeper understanding of lyrics and their evolution can inform further tasks in music information retrieval and recommender systems. For instance, existing user models could be extended to also include the lyric preferences of users, allowing for better capturing of user preferences and intent, and ultimately improving retrieval tasks such as personalized music access and recommender systems.

## Data Availability

The datasets generated and analyzed during the current study are available on Zenodo: https://doi.org/10.5281/zenodo.7740045. The source code utilized for our analyses is available at https://github.com/MaximilianMayerl/CorrelatesOfSongLyrics.
