# Author Correction: Song lyrics have become simpler and more repetitive over the last five decades

**DOI:** 10.1038/s41598-024-62519-9

**Published:** 2024-05-22

**Authors:** Emilia Parada‑Cabaleiro, Maximilian Mayerl, Stefan Brandl, Marcin Skowron, Markus Schedl, Elisabeth Lex, Eva Zangerle

**Affiliations:** 1 Multimedia Mining and Search Group, Institute of Computational Perception, Johannes Kepler University Linz, Linz, Austria; 2 Department of Music Pedagogy, Nuremberg University of Music, Nuremberg, Germany; 3 AI Lab, Human-Centered AI Group, Linz Institute of Technology, Linz, Austria; 4 Department of Computer Science, University of Innsbruck, Innsbruck, Austria; 5 Austrian Research Institute for Artificial Intelligence, Vienna, Austria; 6 Graz University of Technology, Graz, Austria

Correction to: *Scientific Reports* 10.1038/s41598-024-55742-x, published online 28 March 2024

The original version of the Article contained a computing error in the readability descriptor “number of difficult words”. As stated in the originally published Table 1, in a given text the descriptor considered a word with three or more syllables as a difficult word. However, the Authors noted that the descriptor had originally computed a difficult word as word with two or more syllables. The Authors now changed how the descriptor is being computed, so that it considers the correct cutoff value of three or more syllables.

Consequently, in the Results and Discussion section, under the subheading ‘Analysis 1: Evolution of Lyrics and Descriptor Importance’,

“The R^2^ values obtained per genre are 0.0830 for pop, 0.0717 for rock, 0.3374 for rap, 0.2600 for R&B, and 0.1267 for country.”

now reads

“The R^2^ values obtained per genre are 0.0835 for pop, 0.0699 for rock, 0.3340 for rap, 0.2510 for R&B, and 0.1267 for country.”

In the same section,

“Four descriptors are featured in the top descriptors of three genres each (*ratio verses to sections*, *ratio choruses to sections*, *average token length*, *number of difficult words* (words with three or more syllables)).”

now reads:

“Four descriptors are featured in the top descriptors of three genres each (*ratio verses to sections*, *ratio choruses to sections*, *average token length*, *Dugast’s U* (modelling the number of token types as a function of the token count), and *blank line count*.”

and

“While for pop, the top-10 descriptors contain lexical, structural, and rhyme descriptors, rock additionally features a readability descriptor. For rap, country, and R&B, five categories of descriptors are within the top 10.”

now reads:

“While for pop and rap, the top-10 descriptors contain lexical, structural, diversity, and rhyme descriptors, rock features a readability descriptor. For country, five categories of descriptors are within the top 10.”

and

“Interestingly, descriptors measuring the lexical diversity of lyrics are among the top descriptors for rap (*Summer’s S* that essentially captures the ratio of token types and token count; and Measure of Textual Lexical Diversity *MTLD* that captures the average length of sequential token strings that fulfill a type-token-ratio threshold), country (*compression ratio*, i.e., ratio of the size of zlib compressed lyrics compared to the original, uncompressed lyrics; and *Summer’s S*), and R&B (*Summer’s S*). Emotion descriptors only occur among the most important descriptors for country (*positive emotion*) and R&B (*positive emotions* and *anger*). Readability descriptors are among the top descriptors for rock, rap, and notably, the second most important descriptor for R&B (number of *difficult words*).”

now reads:

“Interestingly, descriptors measuring the lexical diversity of lyrics are among the top descriptors for rap and R&B (*Dugast’s U*; and Measure of Textual Lexical Diversity *MTLD* that captures the average length of sequential token strings that fulfil a type-token-ratio threshold), country (*compression ratio*, i.e., ratio of the size of zlib compressed lyrics compared to the original, uncompressed lyrics), and pop (*Dugast’s U*). Emotion descriptors only occur among the most important descriptors for country (*positive emotion*) and R&B (*positive emotions* and *anger*). Readability descriptors are among the top descriptors for rock (*Dale-Chall* readability score, which is computed based on a list of 3,000 words that fourth-graders should be familiar with).”

Additionally, in the Results and Discussion section, under the subheading ‘Analysis 2: interplay of lyrics descriptors, view counts, and release year’,

“For R&B, there is a positive relationship between *release year* and *lyrics view count*: β = 0.30, p = .003; cf. *lyrics view count* for rap in Table 4.”

now reads:

“For R&B, there is a positive relationship between *release year* and *lyrics view* count: β = 0.32, p = .003; cf. *lyrics view count* for R&B in Table 4.”

and

“Differently, for rock, as expected from the outcomes obtained in the multinomial logistic regression, a strong negative relationship between i and i is shown: β = − 1.47, *p* <.001; cf. *lyrics view count* for rock in Table 4.”

now reads:

“Differently, for rock, as expected from the outcomes obtained in the multinomial logistic regression, a strong negative relationship between *release year* and *lyrics view* is shown: β = − 1.47, *p* < .000; cf. *lyrics view count* for rock in Table 4.”

and

“Concerning complexity, this is displayed by the positive β for *compression ratio* (essentially capturing the repeatability of lyrics) shown by both rap and rock (cf. β = 1.15 and β = 0.82, respectively, in Table 4). This indicates that the lyrics of these two genres become easier to comprehend over time, something that can be interpreted as a sign of increasing repetitiveness and, therefore, simplicity. However, the opposite trend is shown for R&B (cf. β = − 0.73, in Table 4), which suggests that the simplification over time might depend on the musical genre; indeed, this descriptor is not relevant neither for pop nor for country. The decline in lyrics’ difficulty observed over time for rap is confirmed by the negative β for *Simple Measure of Gobbledygook* (SMOG) readability measure (in a sample of 30 sentences, words with three or more syllables are counted and used to compute the final SMOG score). This indicates a detriment in complexity concerning the lyric’s readability (cf. β = − 0.64 in Table 4). This contradicts, to some extent, the increasing use of *difficult words*, over time, shown for rap (β = 0.57), while supporting the increase in complexity shown for R&B (β = 2.08); cf. β for *difficult words* in Table 4. This contradiction supports, however, the conclusion extracted from the *compression ratio*, which shows that the lyrics become more repetitive. Thus, it seems that the increase (in absolute terms) of difficult words is only due to repetitions in the lyrics. Differently, when weighting the number of difficult words according to the number of sentences (which is performed when computing *SMOG*), the effect is negative, meaning that in proportion to the number of sentences, the complexity of the text actually decreases with time. The increase in readability over time is also confirmed for rock, as shown by the positive slope for *Dale-Chall readability score* (cf. β = 1.07 in Table 4; Dale-Chall is computed based on a list of 3000 words that fourth-graders should be familiar with. The number of words contained in the list of easy words is counted and used as input to the score computation.).”

now reads:

“Concerning complexity, this is displayed by the positive β for *compression ratio* (essentially capturing the repeatability of lyrics) shown by rap (cf. β = 0.70 in Table 4). This indicates that rap lyrics become easier to comprehend over time, something that can be interpreted as a sign of increasing repetitiveness and, therefore, simplicity. However, the opposite trend is shown for R&B (cf. β = − 0.96, in Table 4), which suggests that the simplification over time might depend on the musical genre; indeed, this descriptor is not relevant neither for pop nor rock nor country. The decline in lyrics’ difficulty observed over time for rap is confirmed by the negative β for *Simple Measure of Gobbledygook* (SMOG) readability measure (in a sample of 30 sentences, words with three or more syllables are counted and used to compute the final SMOG score). This indicates a detriment in complexity concerning the lyric’s readability (cf. β = − 0.57 in Table 4). The increase in readability over time is also confirmed for rock, as shown by the positive slope for *Dale-Chall readability score* (cf. β = 1.18 in Table 4).”

and

“As expected, the results also show that lexical descriptors have a more prominent role in rap, i.e., the musical genre for which lyrics are most relevant. Indeed, when calculating the predictors block-wise across feature types, this is the type of feature showing the highest adjusted R^2^: for rap (0.22), followed by R&B (0.13). Block-wise adjusted R^2^ per genre for each feature type are as follows. For rap: Complexity (0.04), Readability (0.06), Lexical (0.22), Structure (0.10), Rhyme (0.13), Emotion (0.02); for pop: Readability (0.004), Lexical (0.06), Structure (0.02), Rhyme (0.01), Emotion (0.01); for rock: Complexity (0.01), Readability (0.01), Lexical (0.04), Structure (0.03), Rhyme (0.01); for R&B: Complexity (0.01), Readability (0.01), Lexical (0.13), Structure (0.02), Rhyme (0.02), Emotion (0.04); for country: Readability (0.014), Lexical (0.09), Structure (0.02), Rhyme (0.02), Emotion (0.01).”

now reads:

“As expected, the results also show that lexical descriptors have a more prominent role in rap, i.e., the musical genre for which lyrics are most relevant. Indeed, when calculating the predictors block-wise across feature types, this is the type of feature showing the highest adjusted R^2^: for rap (0.22), followed by R&B (0.13). Block-wise adjusted R^2^ per genre for each feature type are as follows. For rap: Complexity (0.04), Readability (0.04), Lexical (0.22), Structure (0.10), Rhyme (0.13), Emotion (0.02); for pop: Readability (0.01), Lexical (0.06), Structure (0.02), Rhyme (0.01), Emotion (0.01); for rock: Readability (0.01), Lexical (0.04), Structure (0.03), Rhyme (0.01); for R&B: Complexity (0.01), Readability (0.01), Lexical (0.13), Structure (0.02), Rhyme (0.02), Emotion (0.04); for country: Readability (0.014), Lexical (0.09), Structure (0.02), Rhyme (0.02), Emotion (0.01).”

and

“This trend is confirmed by the negative relationship between release year and the *Maas* score, a measure for lexical diversity proposed by Maas^56^ (the score models the type-token ratio (i.e., the ratio of the total number of words and the total number of unique terms) on a log scale), shown for all the genres except country (for which this descriptor is not included in the model as it did not show a significant contribution), which indicates that vocabulary richness decreases with time (cf. negative β for *Maas* in Table 4).”

now reads:

“This trend is confirmed by the negative relationship between release year and the *Maas* score, a measure for lexical diversity proposed by H.-D. Maas^56^ (the score models the type-token ratio (i.e., the ratio of the total number of words and the total number of unique terms) on a log scale), shown for all the genres except country, pop, and rock (for which this descriptor is not included in the model as it did not show a significant contribution), which indicates that vocabulary richness decreases with time (cf. negative β for *Maas* in Table 4).”

and

“The trend toward simplicity over time can also be observed in the structure, which shows a decrease in the *number of sections*, most prominently shown for R&B and rock (cf. β = − 0.75 and β = − 0.70, respectively in Table 4); as well as a general increment (except for country) in the ratio between verses to chorus and verses to sections (cf. positive β for *ratio verses to sections* and *ratio chorus to sections* in Table 4). Similarly, the results for rhyme-related descriptors further confirm the tendency towards simpler lyrics over time for all musical genres. This is particularly shown by the increment of the *rhyme percent* in rap in R&B (cf. β = 1.34 and β = 0.68, respectively) and by a detriment in the number of rhyme words (cf. negative β for all the genres), which shows a decline in the rhymes’ variety over time.”

now reads:

“The trend toward simplicity over time can also be observed in the structure, which shows a decrease in the *number of sections*, most prominently shown for R&B and rock (cf. β = − 0.72 and β = − 0.79, respectively in Table 4); as well as a general increment (except for country) in the ratio between verses to chorus and verses to sections (cf. positive β for *ratio verses to sections* and *ratio chorus to sections* in Table 4). Similarly, the results for rhyme-related descriptors further confirm the tendency towards simpler lyrics over time for all musical genres. This is particularly shown by the increment of the *rhyme percent* in rap in R&B (cf. β = 1.20 and β = 0.68, respectively) and by a detriment in the number of rhyme words (cf. negative β for all the genres), which shows a decline in the rhymes’ variety over time.”

and

“For R&B the results show that the content of the lyrics becomes more negative with time, as shown by the increase in concepts related to *anger* and a detriment in *positive emotions* (cf. β = 1.75 and β = − 0.86, respectively, in Table 4).”

now reads:

“For R&B the results show that the content of the lyrics becomes more negative with time, as shown by the increase in concepts related to *anger* and a detriment in *positive emotions* (cf. β = 1.91 and β = − 0.89, respectively, in Table 4).”

Additionally, in the Results and Discussion section, under the subheading ‘Result summary for both studies’,

“Regarding RQ1 (Which trends can we observe when correlating multifaceted lyrics descriptors with temporal aspects in an evolution analysis?), we come to the following conclusion: Despite minor contradictory outcomes concerning complexity and readability for rap and rock in comparison to pop and R&B, the interpretation of the lyric’s lexical component, structure, and rhyme, for all investigated genres, generally shows that lyrics are becoming simpler over time^11^, as shown both analyses. This is shown by a decline in vocabulary richness for some specific genres, i.e., rap and rock, and by a general increase in repetitiveness for all the evaluated musical styles. Besides this, lyrics seem to become more emotional with time for rap, and less positive for R&B, pop, and country. Also, we observe a trend towards angrier lyrics across all genres. Potential reasons for the trend towards simpler lyrics are discussed by Varnum et al.^11^. They speculate that this might also be related to how music is consumed, technological innovation, or the fact that music is mostly listened to in the background. As for RQ2 (Which role does the popularity of songs and lyrics play in this scenario?), we conclude that while song listening counts do not show any effects, lyrics view counts do indeed show effects. This suggests that for rap, rock, and country, lyrics play a more pronounced role than for other genres and that listeners’ interest in lyrics goes beyond musical consumption itself.”

now reads:

“Regarding RQ1 (Which trends can we observe when correlating multifaceted lyrics descriptors with temporal aspects in an evolution analysis?), we come to the following conclusion: Despite minor contradictory outcomes concerning complexity and readability for rap and R&B, the interpretation of the lyric’s lexical component, structure, and rhyme, for all investigated genres, generally shows that lyrics are becoming simpler over time^11^, as shown both analyses. This is shown by a decline in vocabulary richness for some specific genres, i.e., rap and R&B, and by a general increase in repetitiveness for all the evaluated musical styles. Besides this, lyrics seem to become more emotional with time for rap, and less positive for R&B, pop, and country. Also, we observe a trend towards angrier lyrics across all genres except for rock. Potential reasons for the trend towards simpler lyrics are discussed by Varnum et al.^11^. They speculate that this might also be related to how music is consumed, technological innovation, or the fact that music is mostly listened to in the background. As for RQ2 (Which role does the popularity of songs and lyrics play in this scenario?), we conclude that while song listening counts do not show any effects, lyrics view counts do indeed show effects. This suggests that for rap, rock, and country, lyrics play a more pronounced role than for other genres and that listeners’ interest in lyrics goes beyond musical consumption itself.”

Furthermore, in the Results and Discussion section, under the subheading ‘Limitations’,

“Another limitation of the work at hand is the *restriction to English lyrics*. This choice had to be made to ensure a language-coherent sample of songs and, consequently, the comparability of results. While some of the descriptors could have been computed for other languages as well, due to the different characteristics of languages (e.g., different lexical structures), a cross-language comparison of the descriptors would not be meaningful. Also, most of the resources required to compute the readability scores and emotional descriptors are only available for English. Nevertheless, in future work, we could include more languages and conduct analyses on songs within each language class on a limited set of suited descriptors.”

now reads:

“Another limitation of the work at hand is the *restriction to English lyrics*. This choice had to be made to ensure a language-coherent sample of songs and, consequently, the comparability of results. While some of the descriptors could have been computed for other languages as well, due to the different characteristics of languages (e.g., different lexical structures), a cross-language comparison of the descriptors would not be meaningful. Also, most of the resources required to compute the readability scores and emotional descriptors are only available for English. Here we note that for assessing the readability of texts, we rely on rather simple metrics. For instance, difficult words are defined as words with three or more syllables. We also note that some of the readability metrics rely on sentences, which might not always be directly extractable from lyrics. Nevertheless, in future work, we could include more languages and conduct analyses on songs within each language class on a limited set of suited descriptors.”

Finally, the values presented in Tables 3 and 4, and corresponding captions, have been updated. The original Tables 3 and 4, with accompanying captions, appear below.

**Table 3:** Lyric descriptors identified by robust regression (Huber M regressor).

Genre					
	Pop	Rock	Rap	Country	R&B
1	Unique rhyme words^Rh^	Repeated line ratio^L^	Ratio verses to sections^S^	Punctuation ratio^L^	Repeated line ratio^L^
2	Repeated line ratio^L^	Title occurrences^S^	Unique rhyme words^Rh^	Compression ratio^D^	Difficult words^Re^
3	Ratio chorus to sections^S^	Number of verses^S^	Ratio chorus to sections^S^	Tris legomenon ratio^L^	Blank line count^L^
4	Dot count^L^	Avg. token length^L^	Repeated line ratio^L^	Avg. token length^L^	Unique rhyme words^Rh^
5	Ratio verses to sections^S^	Difficult words^Re^	Dot count^L^	Blank line count^L^	Parenthesis count^L^
6	Title occurrences^S^	Dot count^L^	Difficult words^Re^	Summer’s S^D^	Positive emotion^E^
7	Avg. token length^L^	Ratio chorus to sections^S^	Exclamation mark count^L^	Unique rhyme words^Rh^	Rhyme percent^Rh^
8	Parenthesis count^L^	Unique rhyme words^Rh^	Hyphen count^L^	Positive emotion^E^	Summer’s S^D^
9	Pronoun frequency^L^	Dis legomenon ratio^L^	Summer’s S^D^	Ratio verses to sections^S^	Dot count^L^
10	Exclamation mark count^L^	Two verses at least one chorus^S^	MTLD^D^	Number of couplets^Rh^	Anger^E^

Superscripts denote descriptor categories, with L = lexical, E = emotion, S = structural, Rh = rhyme, Re = readability, and D = diversity.

**Table 4:** Results of the best-fitting models for the predictors for predicting the release year, considering each genre individually (rap, pop, rock, R&B, and country). $\beta$ coefficient, standard error (*SE*), and *p*-value are reported.

	Rap			Pop			Rock			R&B			Country		
	$\beta$	*SE*	*p*	$\beta$	*SE*	*p*	$\beta$	*SE*	*p*	$\beta$	*SE*	*p*	$\beta$	*SE*	*p*
Lyrics view count	0.22	0.10	.031				$-1.47$	0.43	${\textbf {.000}}$	0.30	0.10	${\textbf {.003}}$	1.22	0.84	.148
Complexity															
Compression ratio	1.15	0.34	${\textbf {.001}}$				0.82	0.31	${\textbf {.009}}$	$-0.73$	0.25	${\textbf {.004}}$			
Readability															
SMOG	$-0.64$	0.13	${\textbf {.000}}$				0.54	0.26	.035				0.54	0.36	.130
Difficult words	0.57	0.15	${\textbf {.000}}$							2.08	0.41	${\textbf {.000}}$			
Dale-Chall				0.50	0.21	.016	1.07	0.35	${\textbf {.003}}$						
Lexical															
Blank line count				0.28	0.17	.093				$-0.65$	0.19	${\textbf {.001}}$	1.23	0.37	${\textbf {.001}}$
Blank line ratio	$-0.66$	0.18	${\textbf {.000}}$										$-1.31$	0.28	${\textbf {.000}}$
Repeated line ratio	2.56	0.29	${\textbf {.000}}$	1.48	0.20	${\textbf {.000}}$	1.05	0.24	${\textbf {.000}}$	3.19	0.24	${\textbf {.000}}$	0.56	0.21	${\textbf {.008}}$
Exclamation mark count	$-0.42$	0.10	${\textbf {.000}}$	$-0.41$	0.19	.032				$-0.38$	0.26	.140	$-1.73$	0.70	${\textbf {.013}}$
Question mark count	$-0.37$	0.12	${\textbf {.003}}$				0.68	0.30	.022						
Hyphen count	$-0.91$	0.15	${\textbf {.000}}$				0.75	0.25	${\textbf {.002}}$				$-0.78$	0.39	.042
Comma count				1.17	0.23	${\textbf {.000}}$	1.33	0.47	${\textbf {.005}}$						
Parens count	0.56	0.13	${\textbf {.000}}$	$-0.67$	0.22	${\textbf {.003}}$	1.13	0.42	${\textbf {.007}}$	$-0.54$	0.15	${\textbf {.000}}$			
Punctuation ratio							$-1.45$	0.37	${\textbf {.000}}$				1.93	0.36	${\textbf {.000}}$
Stop words ratio	0.44	0.25	.082				0.47	0.21	.025	$-0.88$	0.24	${\textbf {.000}}$			
Stop words per line										1.18	0.34	${\textbf {.000}}$			
Dis legomenon ratio	$-0.83$	0.28	${\textbf {.003}}$				$-0.66$	0.21	${\textbf {.001}}$	$-0.50$	0.20	.014			
Tris legomenon ratio	0.68	0.33	.040										0.85	0.17	${\textbf {.000}}$
Maas	$-1.43$	0.35	${\textbf {.000}}$	$-0.28$	0.15	.064	$-0.54$	0.31	.075	$-1.10$	0.27	${\textbf {.000}}$			
Pronoun frequency	2.25	0.27	${\textbf {.000}}$	0.45	0.16	${\textbf {.005}}$				0.72	0.22	${\textbf {.001}}$	$-0.60$	0.19	${\textbf {.001}}$
Past tense ratio	0.29	0.20	.141				$-0.25$	0.17	.133	0.41	0.19	.033	$-0.56$	0.14	${\textbf {.000}}$
Structure															
Title occurrences							1.32	0.25	${\textbf {.000}}$						
Number of sections	$-0.65$	0.25	.010	$-0.39$	0.25	.117	$-0.70$	0.22	${\textbf {.001}}$	$-0.75$	0.27	${\textbf {.005}}$			
Ratio verses sections	1.54	0.19	${\textbf {.000}}$	0.57	0.23	.015							$-0.84$	0.25	${\textbf {.000}}$
Ratio chorus sections	1.17	0.29	${\textbf {.000}}$	0.98	0.28	${\textbf {.000}}$	0.79	0.23	${\textbf {.001}}$	0.82	0.32	.011			
Alternation verse chorus							$-0.45$	0.16	${\textbf {.005}}$	0.53	0.21	.011	$-0.25$	0.14	.063
Two verses/one chorus	0.78	0.22	${\textbf {.000}}$							$-0.75$	0.26	${\textbf {.004}}$			
Rhyme															
Number alternating	$-0.61$	0.16	${\textbf {.000}}$				$-0.79$	0.22	${\textbf {.000}}$				$-0.81$	0.46	.078
Number nested										1.15	0.42	${\textbf {.007}}$			
Rhyme percent	1.34	0.30	${\textbf {.000}}$							0.68	0.22	${\textbf {.002}}$			
Unique rhyme words	$-1.97$	0.16	${\textbf {.000}}$	$-1.01$	0.22	${\textbf {.000}}$	$-1.28$	0.23	${\textbf {.000}}$	$-1.74$	0.27	${\textbf {.000}}$	$-0.93$	0.22	${\textbf {.000}}$
Emotion (LIWC)															
Anger	0.51	0.13	${\textbf {.000}}$	0.26	0.16	.103				1.75	0.30	${\textbf {.000}}$	0.72	0.32	.025
Sad	0.85	0.30	${\textbf {.004}}$										$-0.38$	0.16	.018
Positive emotions	0.71	0.27	${\textbf {.009}}$	$-0.85$	0.15	${\textbf {.000}}$				$-0.86$	0.13	${\textbf {.000}}$	$-0.71$	0.18	${\textbf {.000}}$

Significance below the threshold of .01 is highlighted. Due to space constraints, we only report fixed effect estimates showing meaningful impact (i. e., $p<.01$) for at least one genre. Adjusted R^2^ for each genre is: rap (0.3), pop (0.08), rock (0.09), R&B (0.2), and country (0.1).

The original Article has been corrected.

